# Genitourinary rhinosporidiosis

**DOI:** 10.4103/0970-1591.42632

**Published:** 2008

**Authors:** Dilip Kumar Pal, Dhrubajyoti Moulik, Manoj Kumar Chowdhury

**Affiliations:** Department of Urology, Bankura Sammilani Medical College, Bankura - 722 102, West Bengal, India; 1 Department of Surgery, Bankura Sammilani Medical College, Bankura - 722 102, West Bengal, India; 2 Department of Pathology, Bankura Sammilani Medical College, Bankura - 722 102, West Bengal, India

**Keywords:** Genitourinary, rhinosporidiosis, urethra

## Abstract

Rhinosporidiosis is a chronic granulomatous condition commonly affecting the anterior nares and nasopharynx. Apart from these two sites, infection in other sites is very rare. Here is a review of rhinosporidiosis in genitourinary organs with addition of five new cases.

## INTRODUCTION

Rhinosporidiosis is a granulomatous disease caused by the fungus *Rhinosporidium seeberi*. The first case of rhinosporidiosis was described as nasal polyp by Guillermo Seeberi from Buenos Aires in 1900 and at that time the organism was considered as sporozoon. The lifecycle of this fungus was described by Ashworth in 1923 and established the name *Rhinosporidium seeberi*.[1] Though it has a global distribution, 90% cases are from Asia, mainly from south India, Sri Lanka and Pakistan and less than 5% cases are from Africa and the western countries.

## CASE REPORTS

### Case 1

**Figure 1:**
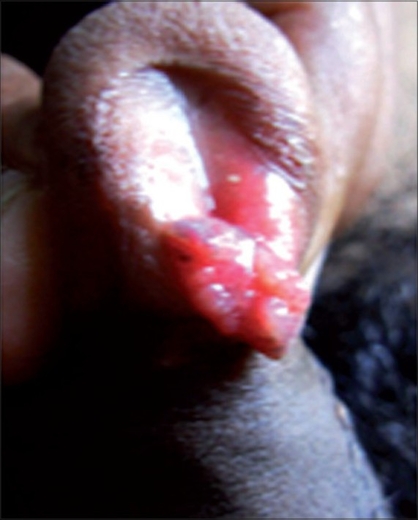
Rhinosporidiosis in terminal male urethra

A male patient, aged 24 years presented with a pinkish polypoidal mass on the external urethral meatus without any obstruction to the urinary stream. Condylomata acuminata was the clinical diagnosis [Figure 1]. Excision of the mass with diathermy coagulation of the base was done. Histopathology suggested rhinosporidiosis of the urethra.

### Case 2

A male patient, aged 36 years presented with postcoital bleeding since last three months. On retraction of the foreskin a pinkish mass with granular surface was noted. After a ventral meatotomy a mass with broad base arising from the fossa navicularis was found. Excision of the mass with diathermy fulguration of the base was done. Histopathology showed it to be a rhinosporidiosis.

### Case 3

A male patient, aged 43 years presented with a slow-growing mass on the glans penis since two years with bleeding from the mass off and on. The mass was on the mucocutaneous junction at the glans penis with nodular surface mimicking carcinoma penis. Biopsy from the lesion showed rhinosporidiosis. Excision of the mass with diathermy cauterization of the base was done. Histology showed it to be rhinosporidiosis.

### Case 4

**Figure 2:**
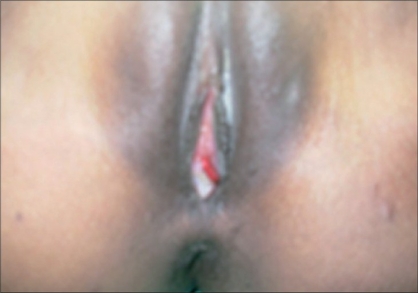
Rhinosporidiosis in female urethra

A female patient aged 23 years, presented with a tongue-shaped projection of a fleshy mass from the posterior lip of the urethra [Figure 2] with a preoperative diagnosis of urethral leiomyoma. Wedge biopsy from the mass showed it to be urethral rhinosporidiosis. Cystoscopy showed no other lesion in the urethra. Adequate excision of the mass with diathermy was done. She developed meatal stenosis and now is on self-meatal dilation without any recurrence for three years.

### Case 5

A male patient aged 27 years, presented with a protrusion of a mass from the urethra during micturition. Pressing the glans penis the tip of a pinkish fleshy mass was seen. Ultrasonography of the penis suggested a mass in the fossa navicularis. The mass was removed after a ventral meatotomy with diathermy fulguration of the base and cystoscopy did not show any other mass. Histopathology suggested urethral rhinosporidiosis.

## DISCUSSION

The disease usually affects mainly the nasal mucosa (70%), nasopharynx (6%) and conjunctiva. The first case of genitourinary rhinosporidiosis was reported by Dhayagude in 1941 from India in the urethra of a male patient.[2] Only a series of 27 cases has been published from India by Sasidharan *et al*. in 1987.[2] Males in their active life are more commonly infected than females. Mostly in all the cases the patients presented from the rural areas. Though the mode of infection is not clearly established, frequent bathing in contaminated stagnant water seems to be the cause of infection on a traumatized mucosa.[2,3] It is not contagious and till now there is no evidence of transmission of the disease from man to man or animal to man.[1,2] The question of sexual transmission was raised by Symmers[5] of vulval rhinosporidiosis in a female and urethral rhinosporidiosis in a male, both of whom were sexual partners, but it has not been documented by any other author including in the present series.

Usually, the lesion presents as a discrete, friable, painless, slow-growing polypoidal pedunculated or sessile mass mainly in the fossa navicularis, with a clinical suspicion of condylomata acuminata or lata. Sometimes it may extend up to the spongy urethra.[3,4] The penile involvement occurs as a polypoidal penile growth in the shaft penis or on the glans penis mimicking penile malignancy. Female urethral involvement is very rare with and the first published report of two cases by Sasidharan.[3] The female case in our series is the third case to our knowledge. She presented herself with a tongue-shaped projection from the posterior urethra with a clinical impression of urethral caruncle. Till date only three female genital rhinosporidiosis have been reported. One in labia minora presented with bleeding from a painful nodule with a preoperative diagnosis of infected retention cyst or endometriosis,[4] one in vulva presented with vulval growth[1] and the third one a 60-year-old lady presented with a pedunculated vaginal growth near the introitus.[5]

**Figure 3:**
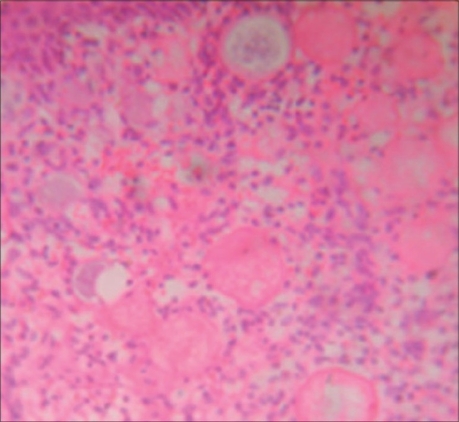
Microphotograph showing sporangia in various stages of maturity with infiltration of chronic inflammatory cells (H and E, ×400)

Histopathology is the only way to diagnose the condition. The typical histology [Figure 3] shows the sporangia in various stages of maturity enclosed in a thick chitinous wall. The overlying squamous epithelium is usually hyperplastic. There is infiltration of chronic inflammatory cells like lymphocytes, plasma cells and polymorphomonuclear leukocytes. Giant cell reactions may be seen near the ruptured sporangia.

Surgical excision of the lesion with diathermy coagulation of the base is the only treatment in rhinosporidiosis.[2,3] Deepseated urethral lesion or multiple lesions require laying open the urethra to ensure complete extirpation of all the lesions and delayed urethral reconstruction.[2]

Endoscopic resection and electro-fulguration of the base is an alternative treatment.[3]

Follow-up is mandatory because of high recurrences with incidences of 3.6 to 25%.[2,3] The recurrences are mainly due to inadequate excision or reinfection.[3]
